# G6PD Deficiency at Sumba in Eastern Indonesia Is Prevalent, Diverse and Severe: Implications for Primaquine Therapy against Relapsing Vivax Malaria

**DOI:** 10.1371/journal.pntd.0003602

**Published:** 2015-03-06

**Authors:** Ari Winasti Satyagraha, Arkasha Sadhewa, Vanessa Baramuli, Rosalie Elvira, Chase Ridenour, Iqbal Elyazar, Rintis Noviyanti, Farah Novita Coutrier, Alida Roswita Harahap, J. Kevin Baird

**Affiliations:** 1 Eijkman Institute for Molecular Biology, Jakarta, Indonesia; 2 University of Northern Arizona, Flagstaff, Arizona, United States of America; 3 Eijkman-Oxford Clinical Research Unit, Jakarta, Indonesia; 4 Centre for Tropical Medicine, Nuffield Department of Medicine, University of Oxford, Oxford, United Kingdom; Walter and Eliza Hall Institute, AUSTRALIA

## Abstract

Safe treatment of *Plasmodium vivax* requires diagnosis of both the infection and status of erythrocytic glucose-6-phosphate dehydrogenase (G6PD) activity because hypnozoitocidal therapy against relapse requires primaquine, which causes a mild to severe acute hemolytic anemia in G6PD deficient patients. Many national malaria control programs recommend primaquine therapy without G6PD screening but with monitoring due to a broad lack of G6PD deficiency screening capacity. The degree of risk in doing so hinges upon the level of residual G6PD activity among the variants present in any given area. We conducted studies on Sumba Island in eastern Indonesia in order to assess the potential threat posed by primaquine therapy without G6PD screening. We sampled 2,033 residents of three separate districts in western Sumba for quantitative G6PD activity and 104 (5.1%) were phenotypically deficient (<4.6U/gHb; median normal 10U/gHb). The villages were in two distinct ecosystems, coastal and inland. A positive correlation occurred between the prevalence of malaria and G6PD deficiency: 5.9% coastal versus inland 0.2% for malaria (P<0.001), and 6.7% and 3.1% for G6PD deficiency (P<0.001) at coastal and inland sites, respectively. The dominant genotypes of G6PD deficiency were Vanua Lava, Viangchan, and Chatham, accounting for 98.5% of the 70 samples genotyped. Subjects expressing the dominant genotypes all had less than 10% of normal enzyme activities and were thus considered severe variants. Blind administration of anti-relapse primaquine therapy at Sumba would likely impose risk of serious harm.

## Author Summary

G6PD deficiency affects over 400 million people worldwide. This enormously diverse disorder causes acute hemolytic anemia upon exposure to oxidizing chemicals, e.g., naphthalene, some sulfa drugs, and certain antimalarials, including primaquine. The primary public health concern with G6PD deficiency involves that latter drug, the only one available for the radical cure of vivax and ovale malarias. Absent primaquine therapy, patients will suffer multiple recurrent attacks called relapses in the two years following the primary attack. Primaquine in G6PD-deficient patients triggers a mild to severe acute hemolytic anemia, depending upon dose administered and the specific variant involved. Relatively high therapeutic doses in severely deficient variants will threaten life. Malaria therapeutic policy and practice regarding primaquine may hinge upon the prevalence and severity of G6PD deficiency weighed against the therapeutic benefit of averting risk of relapse and attendant morbidity, mortality and onward transmission. In the current study we aimed to inform that weighing by characterizing the frequency and type of G6PD deficiency occurring in populations enduring endemic vivax malaria transmission on a single island in eastern Indonesia. The findings infer risk of serious harm caused by primaquine administered to residents of unknown G6PD status.

## Introduction

The majority of people suffering acute malaria caused by *Plasmodium vivax* cannot safely receive primaquine therapy to prevent multiple recurrent attacks called relapses. This extraordinary gap likely explains most of the heavy burdens of morbidity and mortality imposed by this long neglected species of parasite. Misunderstood as relatively harmless over past decades, recent work affirms an often pernicious and sometime fatal course with a diagnosis of *P*. *vivax* malaria [1][2][3][4][5]. This realization awakened concern regarding the long neglect of therapy against relapse due to the problem of primaquine toxicity in patients with an inborn deficiency of glucose-6-phosphate dehydrogenase (G6PD).

Glucose-6-phosphate dehydrogenase deficiency (G6PDd) affects more than 400 million people, or about 8% of the general population of malaria endemic nations [6][7]. This problem hampers the malaria elimination aims employing primaquine as anti relaps therapy. G6PD is an inherited, X-linked recessive trait expressed by any one of many dozens of known single nucleotide polymorphisms (SNPs) impairing the function of G6PD enzyme to an extent ranging from very slightly to almost completely [8]. G6PDd remains silent in almost everyone until exposure to certain infections, foods, chemicals or drugs provokes an acute hemolytic anemia (AHA) ranging from mild and self-limiting to severe and life threatening [9]. The severity of AHA appears to be directly correlated with the extent to which G6PD activity is impaired, and such is the basis of the classification of the many known G6PDd variants put forth by the World Health Organization [10].

The most important clinical and public health problem with G6PDd stems from the hemolytic toxicity of primaquine. This drug stands alone as the only available therapy against both onward transmission of the infection via sexual blood stages (called gametocytes) and in preventing relapses caused by latent forms in the liver (called hypnozoites). Therapeutic doses of primaquine cause a mild to severe AHA, depending upon dose delivered and the G6PDd variant involved, and there is great variation in each in the clinical setting.

Gametocytocidal therapy has been a single adult dose of 45mg primaquine base, whereas hypnozoitocidal therapy has been a daily dose of 15 or 30mg daily for 14 days (210 or 420mg total dose) [11][12]. Even the single 45mg dose provoked a substantial AHA in otherwise healthy adult volunteers with the relatively severe Mediterranean variant of G6PDd [13], but not among those with a mild A- variant common in Africa and African-Americans [14]. That toxicity, along with recognition of good gametocytocidal efficacy at a single 15mg adult dose, prompted WHO in 2012 to recommend the lower dose [15].

Hypnozoitocidal therapy applies much greater amounts of primaquine, and important differences also occur with regard to variant-specific sensitivity to the drug. When otherwise healthy G6PDd A- adult volunteers were exposed to daily doses of 15mg or 30mg primaquine, hemolysis typically did not commence until after the third or fourth day, and the nadir of hematocrit occurred on about day 7 or 8 of dosing [16]. Thereafter hemolysis seemed to cease and subjects recovered normal hematocrit despite continuous daily dosing of 30mg for many weeks [17]. Only older red blood cells (RBC) were destroyed and the younger RBC replacing them could manage continued primaquine dosing. AHA in those subjects was thus considered mild and self-limiting. In stark contrast, similar experiments in adult Mediterranean G6PDd variants showed exquisite sensitivity to daily dosing without induction of even the slightest tolerance of primaquine [17]. Even reticulocytes of those subjects were destroyed by primaquine challenge. Continued dosing would cause severe and threatening AHA. The danger of primaquine therapy thus hinges upon single (gametocytocidal) versus daily (hypnozoitocidal) dosing, and the ability to identify those most at risk of a relatively severe AHA, i.e., patients having severe G6PDd variants, like Mediterranean.

The majority of malaria patients live where G6PDd cannot be even crudely assessed using commercially available qualitative kits [18][19][20]. These kits are expensive, require specialized equipment and laboratory skills, and a cold chain of supply and storage [21]. Consequently, most malaria patients are not offered primaquine therapy due to the danger it poses to a minority of patients. The opportunity to prevent multiple relapses in the coming weeks and months is thus lost. Multiple preventable attacks, typically at least 3 and sometime 10 or more, occur with attendant deepening risk of morbidity, mortality, and onward transmission [22]. The benefit of withholding primaquine therapy for fear of causing harm among unscreened G6PDd patients must be weighed against the risks borne of repeated clinical attacks and new opportunities for onward transmission. Risk versus benefit deliberation of primaquine therapy should be informed by the prevalence and severity of G6PDd in any given population burdened with endemic malaria vivax transmission. The proportion of people vulnerable to primaquine therapy and the extent of their sensitivity to primaquine underpin that weighing.

In the current study, we characterized the epidemiology of G6PDd at three sites on Sumba Island in the malaria-endemic eastern Indonesian archipelago. Those sites represented relatively low to high risk of malaria. We quantitatively assessed G6PD activity in blood from nearly 2000 residents of most ages and both sexes and genotyped those exhibiting impaired G6PD activity. The effort represents a primary step in beginning to grasp the likely clinical consequences of primaquine therapy without G6PDd screening by delineating quantitative G6PD activity and genotype in a population at risk. The study also lays the foundation for quantitative definition of G6PDd diagnostic device performance and pitfalls in anticipation of practical point-of-care screening later becoming available in such settings.

## Materials and Methods

### Ethics Statement

The study has been approved by the Eijkman Institute Research Ethics Commission (Project Number 46, July 29^th^, 2011) and the study has been conducted according to the principles expressed in the Declaration of Helsinki. Written informed consent was obtained from all subjects whose 8 ml of blood were taken. Parents or guardians signed the informed consent for minors.

### Study Site and Population

**Fig 1:**
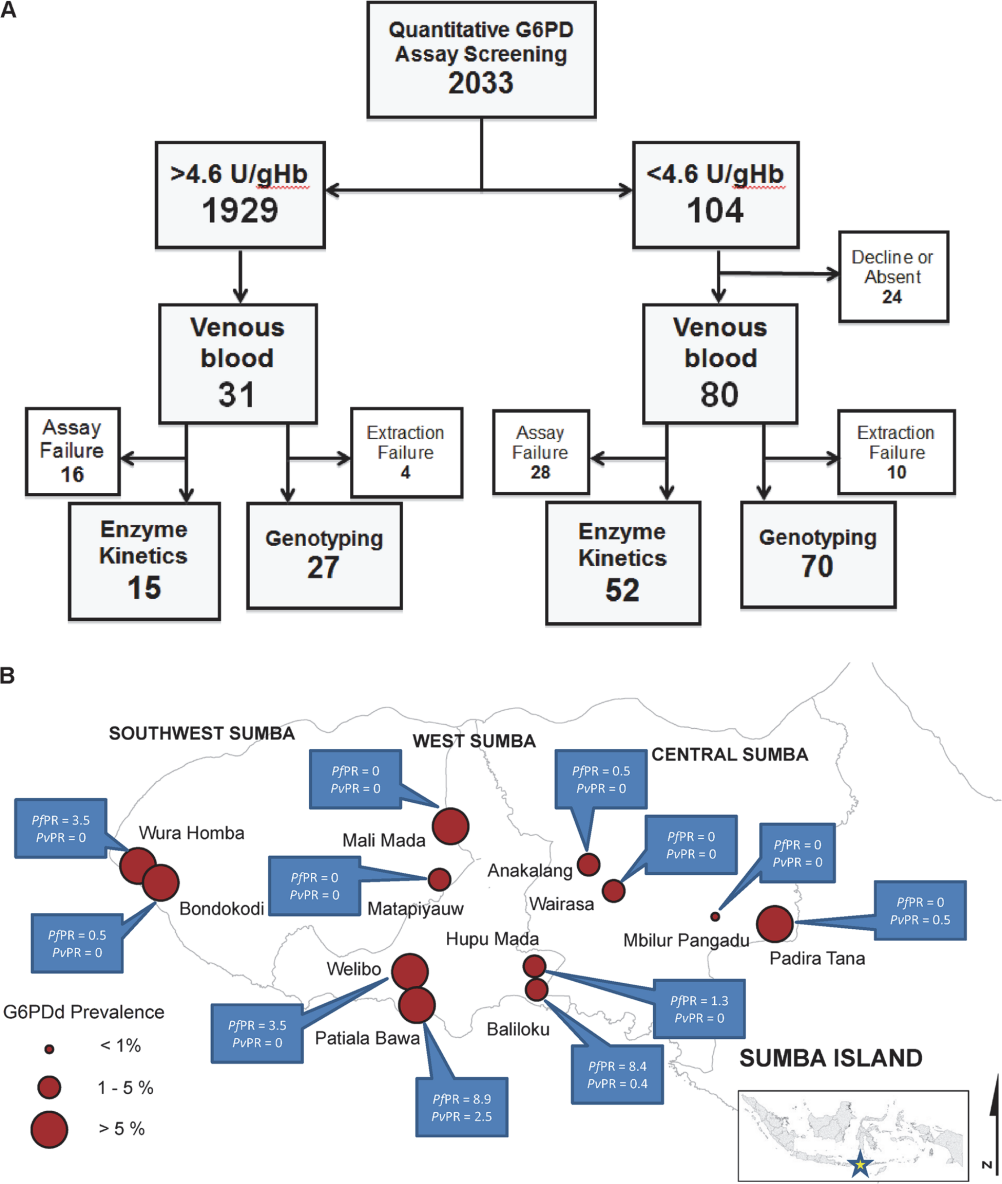
A) Schematic work flow of the study in western Sumba in 2012 and B) map of G6PD deficiency and malaria prevalences in study sites in Sumba. Small inlet showed the map of Indonesia and where Sumba island is located.

Screening of 2033 residents from two different ecosystems (inland and coastal) in western Sumba in the Lesser Sundas Archipelago of eastern Indonesia (Fig. 1) occurred during January-February 2012. The cross-sectional surveys were conducted at three districts in western Sumba comprising inland and coastal ecosystems. The inland ecosystem included Lendiwacu and Wairasa in Central Sumba and Palla health centres in Southwest Sumba whereas the coastal ecosystems included Kabukarodi and Lahihuruk health centres in West Sumba and Bondo Kodi in Southwest Sumba. The sample represented 1–3% of the total population in the areas assessed. The villages sampled represented those in proximity to the health centres from which the research team operated. Most villages were beyond practical reach of sampling that included quantitative G6PD assessments in the field. Residents were invited for screening and the sample thus represents all of those willing to do so.

All volunteers were assessed for malaria, hemoglobin level, and quantitative G6PD activity in an improvised field laboratory utilizing a finger-stick sample of blood in EDTA micro-tubes. Subjects were classified as G6PD normal or deficient by employing 4.6 U/gHb G6PD as cut off point as per recommendation of the quantitative assay manufacturer’s instructions. All G6PDd subjects and 31 G6PD normal subjects who were available on the day of blood collection, having at least 10g/dL hemoglobin (Hb) and being more than 6 years of age were invited to submit to venipuncture (8mL whole blood) for assessment of purified G6PD enzyme kinetics and genotyping at a laboratory in Jakarta. Among the 104 G6PDd residents identified, only 80 volunteered to submit to venipuncture and provided written informed consents, the balance of 24 people were either absent or declined.

### Finger Stick Blood Processing, Parasitological and Hemoglobin Examination and G6PD Assay

Finger stick blood was first placed on a clean glass slide and prepared for thin and thick blood film staining by Giemsa in the standard manner. Certified malaria microscopists read at least 100 visual fields of oil immersion (1000X) magnification of the stained thick film prior to considering a slide negative. Positive slides were reported according to species observed in the microscopic examination from thin blood film. Blood from finger stick of volunteers was placed in EDTA micro-tubes (Becton-Dickinson Microtainer), placed in a cool dark container, and within 8hr was quantitatively assayed for G6PD activity using a commercially available kit (Trinity Biotech, Ireland; Cat. No. 345-B) with deficient (Cat. No. G5888), intermediate (Cat. No. G5029) and normal (Cat. No. G6888) G6PD controls. The reaction was read at 340nm wavelength using a Biochrom (UK) WPA Biowave II UV/Vis spectrophotometer. G6PD activity was calculated from that optical density reading as instructed by the kit manufacturer. Ten microliters of EDTA blood from the microtube was put into a micro-cuvette supplied by the manufacturer of the HemoCue system (HemoCue AB, Sweden) and immediately read in the instrument (Hb201+) of that system for hemoglobin measurement prior to the G6PD quantitative assay.

### Venipuncture Blood Processing

A qualified phlebotomist collected 8mL venous blood from the arm into 8.5mL vacuum capped tubes containing acid citrate dextrose (ACD; Becton Dickinson yellow top vacutainer). These tubes were immediately stored at 4°C for no more than 5 days in Sumba prior to transport by air to the laboratory in Jakarta. The tubes were also stored at 4°C in the laboratory until the day of processing. All blood was analyzed within 30 days of collection.

Venous blood was processed for genotyping and G6PD purification and enzyme kinetics assays. After being spun 1500xg for 15min, the plasma was decanted by pipette, followed by transfer of the buffy coat into a 1.5mL Eppendorf tube held at −25°C for later genotyping. The red blood cell (RBC) pellet was then washed with isotonic solution (0.9% NaCl with 1mM EDTA) and spun at 500xg for 15min 3 times before adding lysis solution (2.7 mM EDTA with 0.7 mM β-mercaptoethanol) in 1:5 ratios. The hemolysate was then centrifuged at 20,000xg for 30 min and the supernatant poured slowly into the DEAE column (GE Healthcare, USA) equilibrated with Buffer 1 (5 mM Na-phosphate buffer, pH 6.4). After lysate was loaded to this column, Buffer 2 (5 mM Na-phosphate buffer, pH 6.4 containing: 1mM EDTA, 20 μM NADP, 1 mM β-mercaptoethanol) was added. Partially purified G6PD was eluted from the column using Buffer 3 (0.1 M Na-phosphate Buffer pH 5.8 containing: 0.5 M NaCl, 1 mM EDTA, 20 μM NADP, 1 mM β-mercaptoethanol). G6PD activity of this crude eluate (DEAE fraction) was measured prior to being put through a 10 KD Milipore size-exclusion chromatography spin column to concentrate and to exchange Buffer 3 with ADP Wash Buffer 1 (0.1M K-acetate + 0.1M K-phosphate pH 6) ready for next purification step. That eluate (Millipore fraction) was then put into 2’,5’ ADP-Sepharose affinity chromatography column (BioRad, USA) and the column was washed in ADP Wash Buffer 2 (0.1M K-acetate + 0.1M K-phosphate pH 7.85), ADP Wash Buffer 3 (0.1M KCl + 0.1M K-phosphate pH 7.85), and finally with Equilibration Buffer (50nM K-phosphate buffer containing 1 mM EDTA pH 7.5) all with flow rate of 50ml/hr. The elution buffer (80mM K-phosphate, 80mM KCl, 1 mM EDTA pH 7.85 and 0.4mM NADP^+^) was added and fractions 4 and 5 (1 ml each), containing purified G6PD from 2’5’ ADP-Sepharose affinity chromatography, were stored on ice no more than 4hr prior to biochemical characterization. This enzyme purification method was adapted from protocols describe elsewhere [23][24].

### Enzymatic Activity, Kinetics of Purified G6PD

In each tube, 0.2mM NADP^+^, 0.1M Tris HCl pH 8.0, 0.01M MgCl_2_ and purified enzyme from fractions 4 and 5 of 2’5’ ADP-Sepharose affinity chromatography (diluted 1:5 with lysis solution) was incubated for 10 min at 30°C. Water (blank) or substrate (0.6mM glucose-6-phosphate, G6P) was then added to the mixture and absorbance read at 340nm using the 25 Lambda™ UV/Vis spectrophotometer (Perkin Elmer, USA) for 5 min with 1 min interval for optical density (OD) readings. The calculation of activity was as follows:

$$\text{Activity}\;\left(\text{U}\right)=\frac{\Delta OD/\text{min}\times 10^{5}}{6.22\times \text{enzyme volume}}$$

(enzyme volume = μl of enzyme/ml reaction mixture) where one unit of G6PD consists of the amount of enzyme which reduces one μmol of NADP^+^ per minute. One μmol/min of reduced NADP (NADPH) has an absorbance of 6.22 in a light path of 1 cm.

Five different concentrations of NADP^+^ (0.01mM, 0.02mM, 0.03mM, 0.04mM and 0.05 mM) and constant G6P concentration or 5 different concentrations of G6P (0.03mM, 0.06mM, 0.12mM, 0.18mM and 0.16mM) with constant NADP^+^ and all reactions used constant amount of enzyme pooled from fraction 4 and 5 of 2’,5’ ADP-Sepharose affinity chromatography column [10]. These activities were then measured at 340 nm wavelength as described above and used to calculate enzyme activity.

### G6PD Genotyping

The frozen buffy coat from venous blood samples was thawed and its DNA was extracted using Qiagen Flexigene DNA extraction kit according to the manufacturer’s instruction. PCR of the G6PD gene was done essentially as described by Saunders et al [25]. The gene was divided into 3 segments in which internal primers were designed to amplify the entire gene starting from exon 3 to 13. Primers (ordered from 1stBASE) for sequencing were designed internally from each of the 3 parts and employed a primer walking sequencing strategy [25]. Purifications of the PCR products with High Pure PCR Product Purification Kit from Roche were done prior to cycle sequencing. Sequencing reaction was performed by using the ABI Prism BigDye Terminator cycle sequencing ready kit version 31 and run on 3130 XL genetic analyzer (Applied Biosystems, France). Electropherograms were visualized and analyzed with FinchTV. Nucleotide sequences were compared to the sequence of G6PD in GenBank (accession no. NG_009015.1) for mutation identification.

### Statistical Analyses

Subjects were classified as normal or deficient on the basis of Trinity Biotech’s quantitative G6PD activity above 4.6 U/gHb as normal G6PD. For those with G6PD deficiency, their enzyme activities (phenotype) and G6PD mutations (genotype) were measured. The primary outcome was the prevalence of people in the sample with G6PD deficiency. The result was stratified by study site and gender. Statistical significance of G6PDd prevalence by site and gender was evaluated by Chi-square test. Odd ratios and Fisher’s exact 95% confidence intervals were used to determine the relationship between haemoglobin, gender and parasitemia on deficient phenotype. Mean, median, standard deviation and range of G6PD enzymatic activities were calculated to determine reference values in normal and deficient subjects. An extended Wilcoxon rank-sum test was used to evaluate the trend of G6PD activities across anemia and normal Hb level. Data were analyzed using Stata 9.

## Results

### Demography, Parasitology, G6PD Prevalence and Genotypes

In total we covered the villages surrounding the six health centres from these three districts, with three health centres per ecosystem. Among the screened 2,033 subjects, 58% were female at both habitat types. The median age at the inland sites was 29 and 33 years of age (males and females which ranged from 5–76 years and 4–80 years, respectively), older than at the coastal sites (median 16 and 26 for males and females which ranged from 3–80 years old and 3–78 years old respectively) as summarized in Table 1. Hemoglobin levels were essentially similar between inland and coastal sites, with females consistently showing higher rates of anemia. The prevalence of G6PDd phenotype among males at the coastal sites was significantly higher than among those at inland sites (10.8% vs. 3.6%; OR = 3.2; 95%CI = 1.7–6.4; P<0.0001). Among females, no such difference occurred (4.2% vs. 3.0%; OR = 1.4; 95%CI = 0.7–2.8; P = 0.282). Among the 70 G6PDd subjects successfully genotyped, 32 were Vanua Lava (17303T→C), 22 Viangchan (19451G→A), 15 Chatham (19583G→A), and 1 Kaiping (20316G→A).

**Table 1:** Demographic, malaria and G6PDd prevalence data by gender and ecosystem in western Sumba.

Parameter	Inland			Coastal		
	Male	Female	Total	Male	Female	Total
	(n = 417)	(n = 595)	(n = 1012)	(n = 427)	(n = 594)	(n = 1021)
Total population in the area^1^ (n)	19315	18610	37925	41798	37836	79634
Proportioned included in the study (%)	2	3	2.7	1	1.6	1.3
**Age (n, %)**						
< 10	74 (18)	61 (10)	135 (13)	113 (26)	102 (17)	215 (22)
10–20	116 (28)	108 (18)	224 (22)	126 (30)	154 (26)	280 (27)
> 20	226 (54)	426 (72)	652 (65)	188 (44)	337 (57)	525 (51)
Median age	29 (5–76)	33 (4–80)	32 (4–80)	16 (3–80)	26 (3–78)	25.5 (3–80)
**Subject with malaria** ^**2**^ **(n, %)**	1 (0.2)	1 (0.2)	2 (0.2)	30 (7)	30 (5.1)	605.9)
*P*. *falciparum*	-	1	1	27	27	54
*P*. *vivax* ^3^	1	-	1	2	3	5
Mix	-	-	-	1	-	1
Median Hb	13 (6–17.5)	12.3 (3.3–20.6)	12.6 (3.3–20.6)	12.7 (2.8–17.7)	12.1 (3.3–18.6)	12.3 (2.8–18.6)
Subject with anemia^4^	17 (4.1)	46 (7.7)	63 (6.2)	21 (4.9)	48 (8.1)	69 (6.8)
**Subject with G6PDd** ^**5**^ **(%)**	16 (3.8)	15 (2.5)	31 (3.1)	45 (10.5)	23 (3.9)	68 (6.7)
Vanua Lava	6	7	13	12	4	16
Viangchan	3	4	7	6	6	12
Chatham	2	1	3	8	1	9
Kaiping	-	-	-	1	-	1
Not genotyped^6^	5	3	8	19	12	31

n = sample number.

^1^ Population number was obtained from central agency statistic from each district.

^2^ Microscopy diagnosed.

^3^ One subject experienced mixed infection of *P*. *falciparum* and *P*. *vivax*.

^4^ Hb < 10 g/dl.

^5^ G6PD activities ≤4.6 U/gHb.

^6^ Samples screened as G6PD deficient but declined, absent or failed to be DNA extracted.

The prevalence of malaria differed sharply between the two habitat types as shown in Table 1. At the inland sites only 0.2% of the sample was parasitemic compared to 5.9% among coastal residents (P<0.0001). Likewise, the prevalence of of G6PDd among males also differed; 3.8% (16/417) and 10.5% (45/427), respectively (OR = 0.33, 95%CI = 0.18–0.59). The risk of parasitemia among the G6PD normal was slightly lower than among G6PDd, but not significantly different; 3.0% vs.4.8% (OR = 0.60, 95%CI = 0.23–1.54; P = 0.29).

### G6PD Activity in the Community

**Fig 2:**
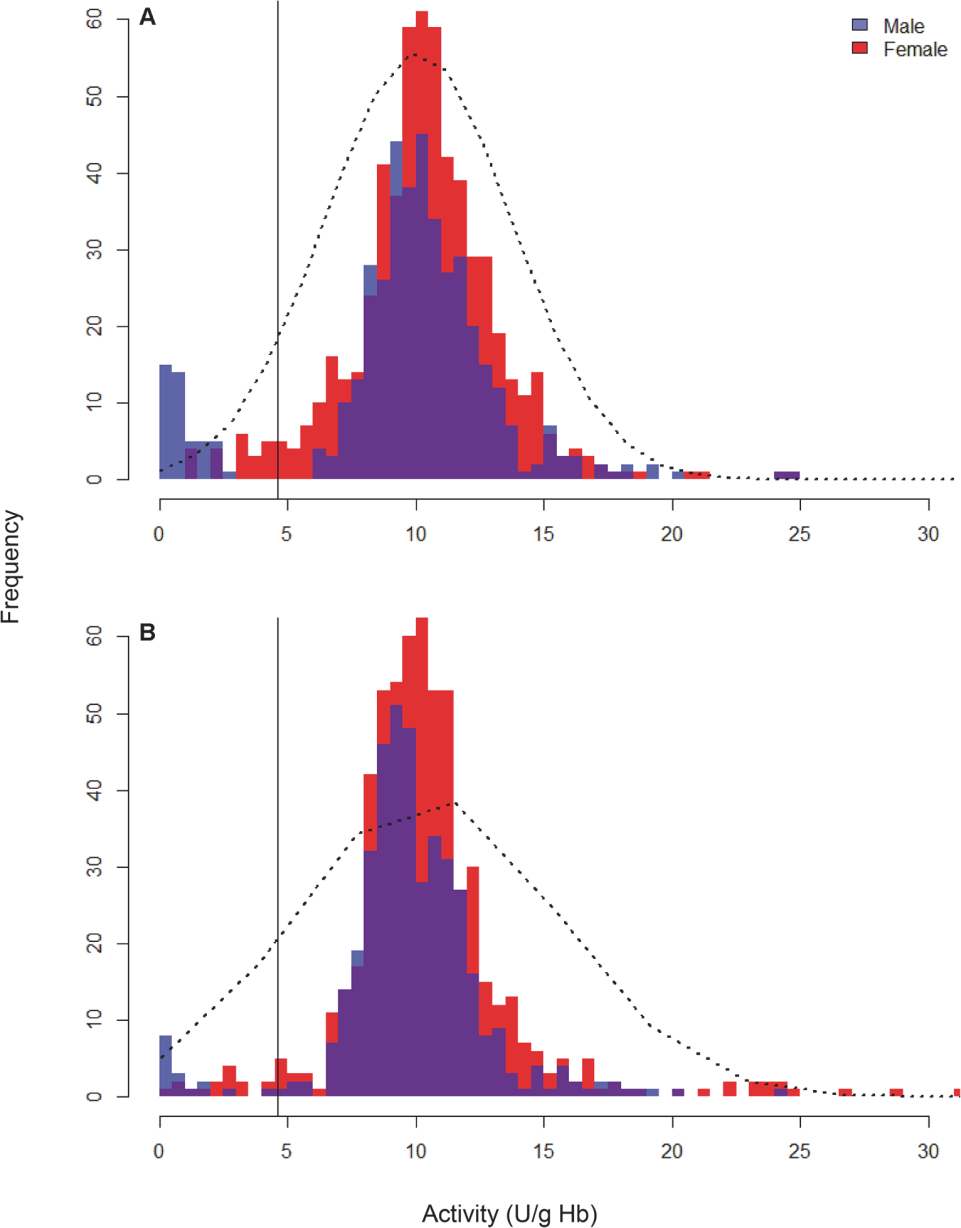
G6PD activity distribution curves in both males (blue) and females (red) in 2 different ecosystems. Black line in both graphs represents the 4.6 U/g Hb as cut off from the Trinity quantitative test manual. A) represents coastal and B) inland areas of western Sumba.

Fig. 2 shows the distributions of G6PD activity values at each of the 2 different ecosystems. Visually, the manufacturer’s recommended cut off for deficient versus normal G6PD activity appears valid. The tail of the normal distribution of G6PD activity approaches a frequency of zero at about that point at both locations. Below that level, the frequency of subjects rises, in most instances to the highest frequency at or near the lowest increment of enzyme activity (0–0.5 U/gHb). Put another way, G6PD activity below about 45% of normal represented the classification of deficient, and the majority of these measurements in men were <10% of normal. Fig. 2B showed that the 45% of normal cut off was even more distinct in inland compared to coastal (Fig. 2A) populations. Table 2 summarizes the essential statistics of G6PD activity among normal and deficient subpopulations with Hb ≥ 8g/dL, respectively, and for male and female subpopulations within each. Normal male G6PD activity ranged from 4.1–47.7 U/gHb and female G6PD activity ranged from 4.6 to 129.9 U/gHb compared to deficient male and female G6PD activities ranged from 0–2.83 U/gHb and 0.46–8.12 U/gHb respectively. The sample with 8.12 U/g Hb had been selected as a normal control for female, however gene sequencing revealed this person was heterozygous for Vanua Lava mutation. The value of 4.1 U/gHb in one normal male was later classified as falsely deficient in the field quantitative test since this individual showed no mutation by sequencing analysis. In this subject both genotyping and enzyme kinetics were wild type and normal (11.61 U/gHb), respectively.

**Table 2:** G6PD activities in normal and deficient subjects by gender in western Sumba.

Parameter	Normal			Deficient		
	Male	Female	Total	Male	Female	Total
**Subject examined**	784	1145	1929	60	44	104
**G6PD enzymatic activity**						
Median	10.2	10.4	10.3	0.9	2.8	1.6
(95% CI)	(10.0–10.3)	(10.3–10.6)	(10.2–10.4)	(0.7–1.1)	(2.4–3.2)	(1.3–1.9)
G6PD Activity in Malaria infection*						
*P*. *falciparum* (n)	10.5 (26)	10.4 (24)	10.5 (50)	0.6 (1)	3.2(3)	2.4(4)
(95% CI)	(9.7–11.3)	(9.4–11.6)	(9.8–11.1)		(1.4–4.1)	(0.6–4.1)
*P*. *vivax* (n)	9.9 (2)	12.0 (2)	10.5 (4)	-	3.2 (1)	3.2 (1)
(95% CI)	(8.5–11.7)	(9.2–14.8)	(8.5–14.8)			
Mix (*Pf* and *Pv*)	0.1 (1)	-	0.1 (1)	-	-	-

SD: standard deviation; all activities are measured in U/g Hb; CI: Confidence Interval

*Median of G6PD activities

**Fig 3:**
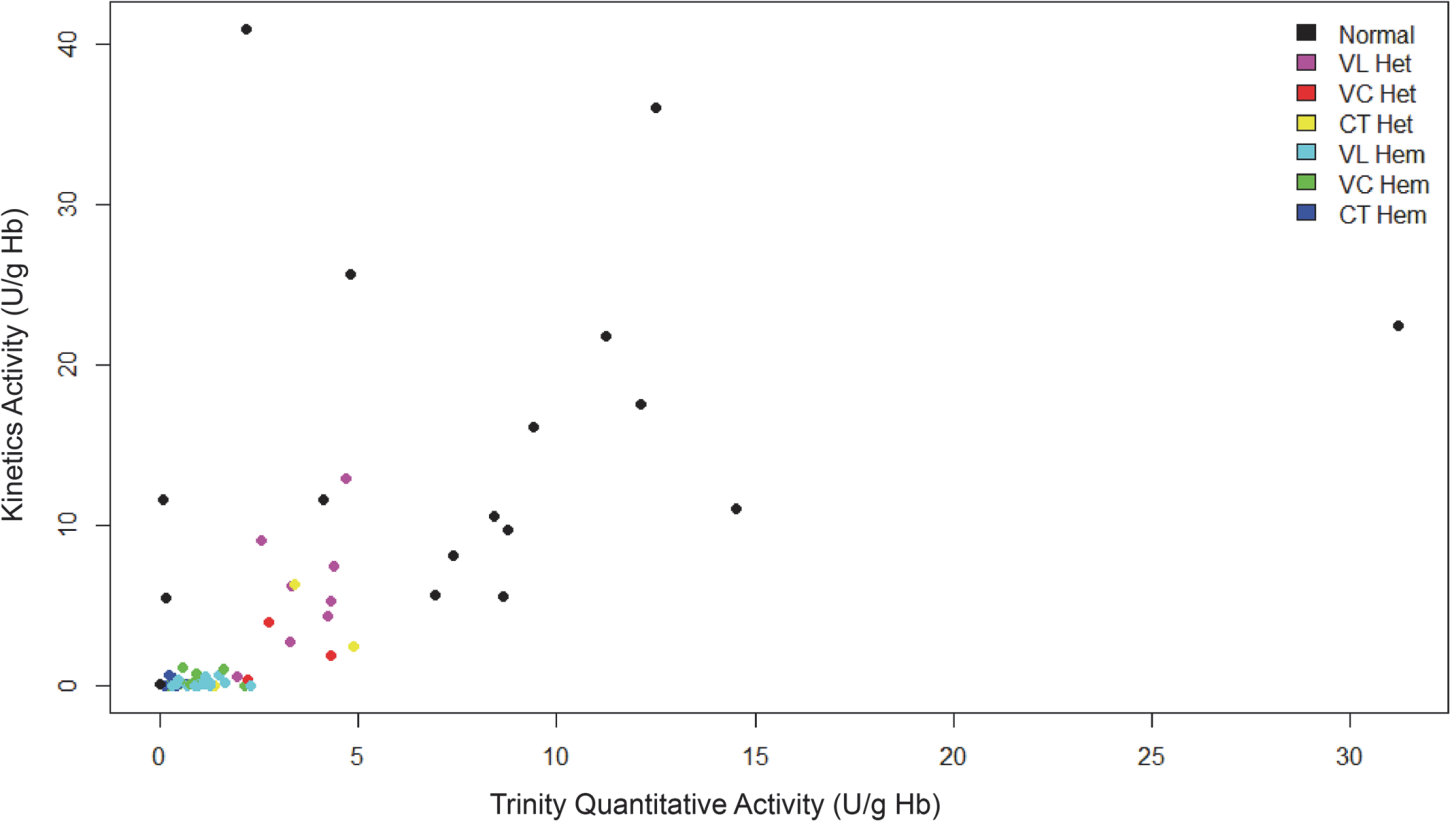
Scatter plot of G6PD activities of G6PD deficient and normal subjects from Trinity quantitative versus kinetics assays measured in U/g Hb. The different colours represented different variant (V in either normal, heterozygous and hemizygous mutants. VL Het, VC Het and CT Het represented heterozygous females having Vanua Lava, Viangchan and Chatham variant respectively. VL Hem, VC Hem and CT Hem represented hemizyogous males having Vanua Lava, Viangchan and Chatham variant respectively.

Fig. 3 illustrates the statistical summary of these measurements according to genotype and zygosity in scatterplot. The normal median of 10.6 U/gHb came with a great deal of variance skewed to higher G6PD levels. Relative to the mostly balanced variance among all classes of G6PDd summarized, the G6PD normal class is relatively very wide and imbalanced. This may be attributed to the outliers seen to the right of the normal distributions of Fig. 2. Variance in G6PD activity among the three dominant genotypes observed (Vanua Lava, Viangchan, and Chatham; VL, VC, & CT, respectively) was very slight among the hemizygous males but was comparatively prominent among heterozygous females especially for VL (Fig. 3). The median G6PD activity among hemizygotes for VL, VC, and CT was 0.13, 0.27, and 0.12 U/gHb, respectively. These are approximately 1% to 2% of normal G6PD activity. The G6PD activities for heterozygous females of these genotypes were 5.3, 1.9, and 2.4 U/gHb for VL, VC, and CT, respectively. These medians ranged from 18% to 52% of normal G6PD activity. Note that the 95% confidence intervals for these heterozygous females all exceed 50% of normal G6PD activity at the upper end, and all reach below 8% at the lower end.

### Impact of Anemia on G6PD Activity

**Fig 4:**
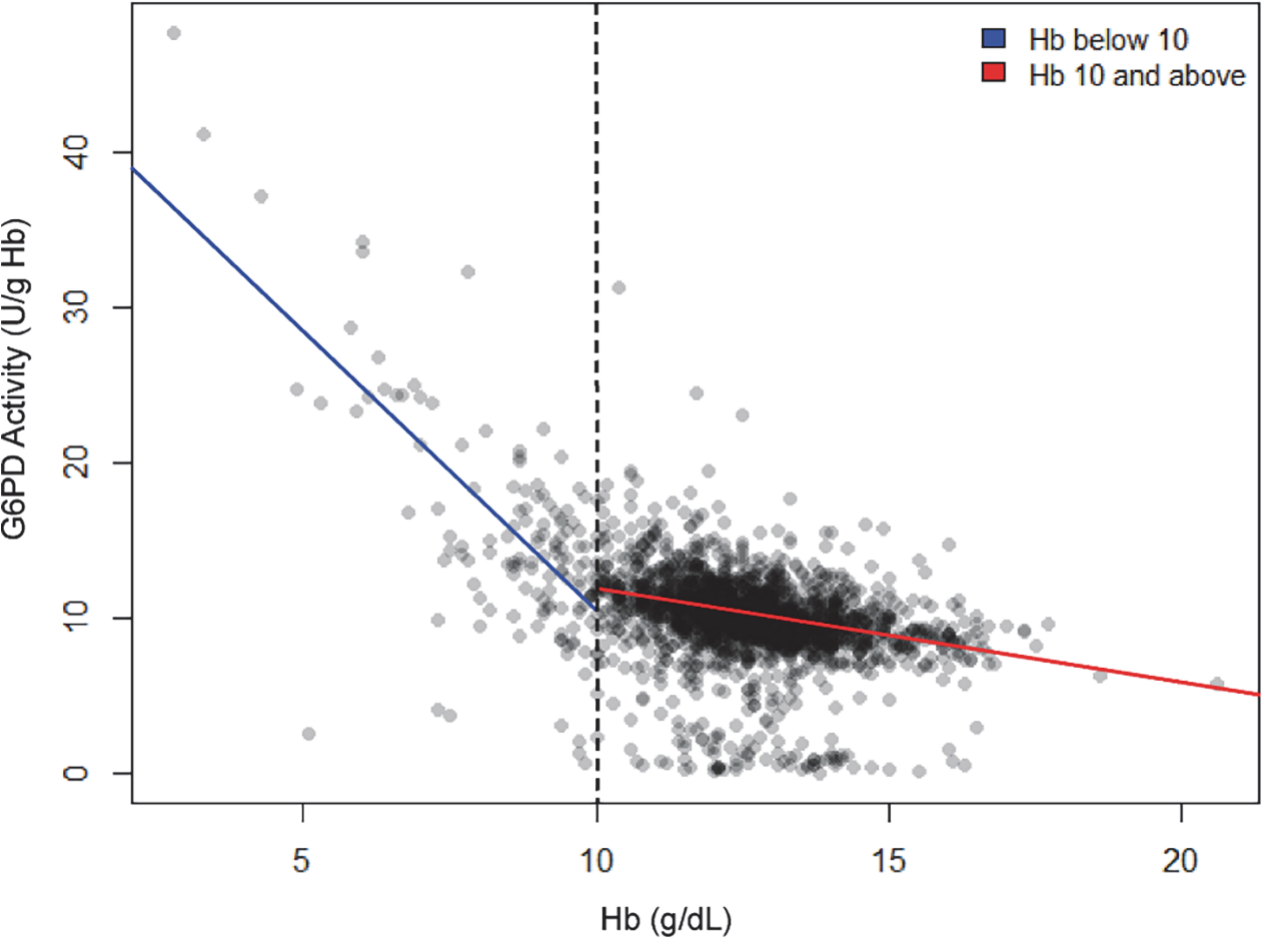
Impact of Hb level on G6PD activities. Blue line represents those samples having extremely high G6PD activity and some degree of anemia (R^2^ = 0.08) and red line represents those having normal Hb level (R^2^ = 0.48) and t-value = 28.6 (p<0.001).

**Fig 5:**
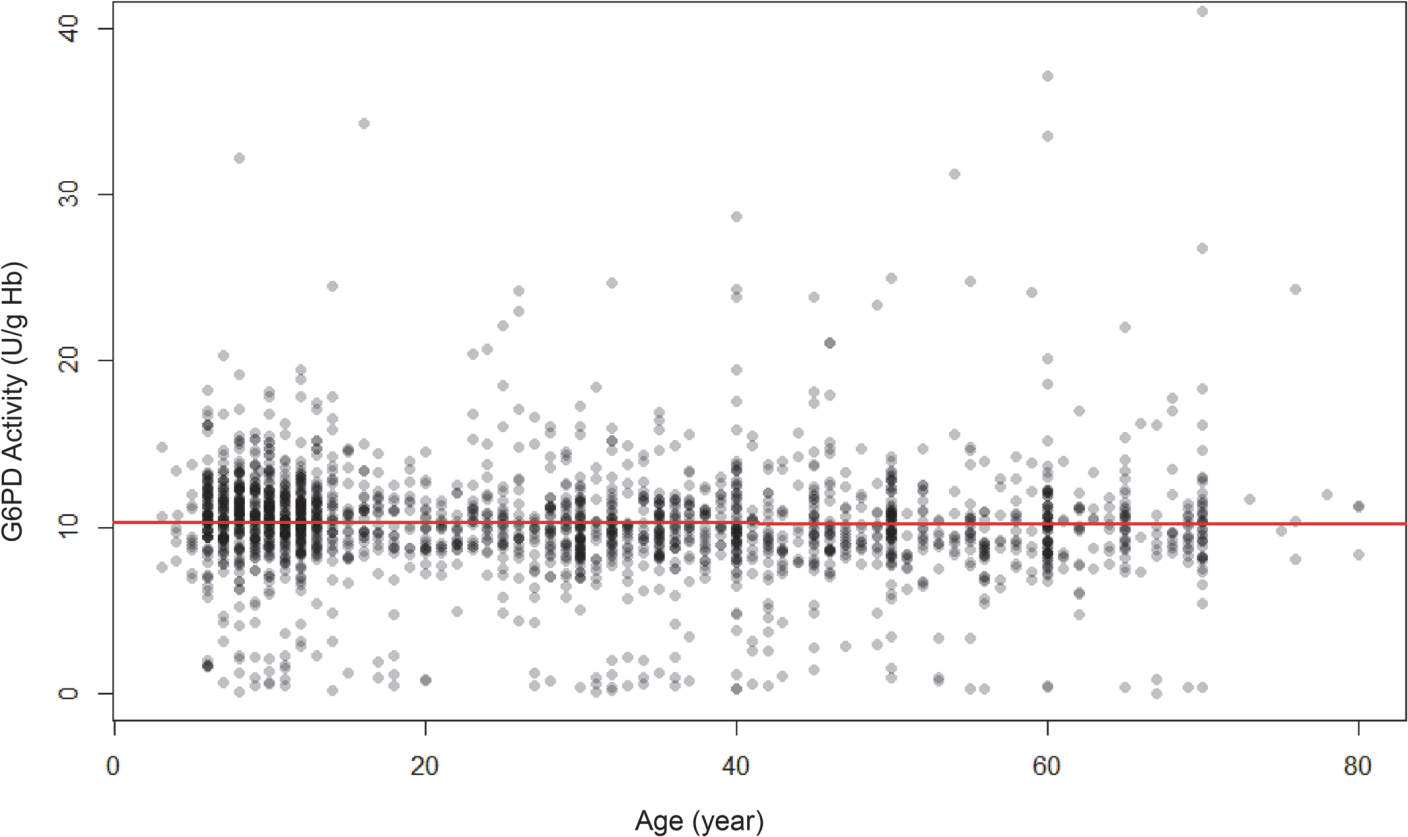
Scatter plot showing the relationship between age and G6PD activity in Sumba.

Hb level as a covariate of G6PD activity is illustrated in Fig. 4. We considered subjects having Hb levels less than 10g/dL as at least moderately anemic. The cluster of normal Hb between 10 and 15g/dL also falls along the G6PD activity norm of about 10 U/gHb. The minor cluster below, representing the G6PDd residents, also scatters evenly between 10 and 15g/dL Hb. The G6PD activity trend line for all of the Hb values above 10g/dL is relatively flat, i.e., apparently not impacting or biasing G6PD activity measurements. In stark contrast, below 10g/dL the trend line rises sharply with diminishing Hb levels, i.e., increasingly severe anemia. Although represented by relatively few data points, the trend was statistically significant (P<0.001). Age may bear upon risk of anemia, and could possibly account for the trend observed, but when G6PD activity was plotted as a function of age (Fig. 5), no trends emerged. Anemia alone profoundly impacted observed G6PD activity measurements.

### Purified G6PD Activity and Michaelis Constants

Table 3 summarizes the enzymatic analyses conducted on purified G6PD from study subjects. The first column from the left lists normal K_m_ values for G6P substrate and NADP^+^ cofactor, along with activity of the purified enzyme. These results, though based on relatively few samples (listed), essentially agreed with the quantitative results derived from hemolysate already detailed. In other words, hemizygous males ranged from 1% to 3% of normal G6PD activity, whereas heterozygous females ranged from 13% to 35% of normal. Low K_m_ for NADP in all variants except CT indicated resistance to inhibition by NADPH and conversely high K_m_ for NADP indicated strong competitive inhibition by NADPH which rendered the variant enzyme to be scarcely functional [26]. High K_m_ for G6P normally resulted in little residual activity in the red cells but does not necessarily mean chronic hemolysis. However, K_m_ values for G6P in hemizygous males for all 3 variants are well below that of normal in comparison to heterozygous females showing close to normal K_m_ for the substrate.

**Table 3:** Enzymatic parameters from purified G6PD from study subjects in western Sumba according to genotype.

	Normal	Heterozygotes			Hemi/homozygotes		
		VL	VC	CT	VL	VC	CT
Sample (n)	14	10	3	2	16	10	9
G6PD (U/gHb)* (95% CI)	14.04 (9.3–20.1)	5.9 (2.8–9.6)	2.05 (0.4–4.0)	4.4 (2.4–6.3)	0.24 (0.1–0.5)	0.28 (0.1–0.9)	0.30 (0.08–0.5)
*K*_m_ G6P(mM)*(95% CI)	0.28 (0.08–1.6)	0.08 (0.04–0.7)	<0.01 **	0.08 (0.06–0.1)	0.06 (0.03–0.1)	0.04 (0.03–0.05)	0.03 (0.02–0.04)
*K*_*m*_ NADP^+^ (mM)* (95% CI)	0.005 (0.004–0.012)	0.008 (0.004–0.03)	0.001 **	0.05 (0.003–0.09)	0.004 (0.001–0.03)	0.001 (0.0003–0.002)	0.0009(0.0005–0.61)

* median of the samples analyzed;

**cannot be calculated because the interval between the data are too small;

CI is confidence interval

All the kinetics parameters (K_m_ for G6P and NADP) as well as the hemolysate enzymes activities showed that the 3 dominant variants found in Sumba have very low activity in the red cell which was in line with reported K_m_ values for each variant. These K_m_ values generally reflect on the characteristic of the enzyme variant *in vivo*. Similarly, pH optimum for each hemizygous variant was found to be in line with the reported values for each (VL is between pH 8.5–9.5, VC is between pH 8.0–9.5 and CT is between pH 9.0–10.0). However, to further characterize the enzyme according to the genotype, further biochemical experiments such as electrophoretic mobility and thermostability were needed, which were not feasible in this study due to time limitation and limited amount of extracted enzyme

## Discussion

The findings reported here corroborate smaller G6PDd surveys done at various locations on Sumba [27][28]. The diversity of G6PDd variants surprises in light of the ethnic homogeneity of people native to Sumba and presumed island founder effect [29][30][31]. It thus seems likely that they acquired that diversity prior to settling Sumba about 30 000–10 000 years ago [32]. In contrast, the Khmer people of Cambodia typically have higher prevalence of G6PDd and over 95% of it represented by Viangchan variant alone [33][34]. Our findings cannot be directly extrapolated to any of Indonesia’s many hundreds of other ethnic groups, but the limited data now available also suggest relatively diverse G6PDd variant representation among them [27][35][36][37][38][39][28]. The apparent diversity of G6PDd in Indonesia imposes difficulties for those evaluating risk of primaquine therapeutic policy and practice in the nation. Understanding this may be illustrated by exploring the implications to such made evident by the observations from Sumba. Each of the three dominant variants among hemizygous males expressed relatively very low residual G6PD enzyme activity—as low or lower than typically reported for the exquisitely primaquine-sensitive Mediterranean variant [40]. We would conclude that all G6PDd males resident in western Sumba would also be exquisitely sensitive to primaquine anti-relapse therapy (not necessarily gametocytocidal therapy) in terms of risk of AHA. Indeed, one male G6PDd subject enrolled in a study executed at western Sumba experienced a steep hemolytic crisis after being dosed for 5 days with 30mg primaquine daily following his misclassification as G6PD normal (Syafruddin D, personal communication). A baseline Hb of 12.4 g/dL registered at 7.2g/dL at clinical assessment, and then 5.6 g/dL at admission to hospital a few hours later. After six days in hospital and blood transfusion therapy, he completely recovered. Close clinical monitoring by the research team had averted deeper harm to that research subject. The blind administration of primaquine anti-relapse therapy on Sumba, in light of our findings and that event and the reality of impractical and improbable clinical monitoring in routine practice, would appear to incur significant risk of serious harm. Screening for G6PDd prior to primaquine therapy would likely be required to protect patients diagnosed with vivax malaria on Sumba.

The heterozygous females impose greater complexity to consideration of primaquine therapy and its safety even with G6PDd screening. The random inactivation of one or the other X chromosome during embryonic development (Lyonization) results in individual females having populations of RBC expressing G6PDd in fixed proportions ranging between 0% to 100%. This may be visualized most clearly among the Vanua Lava heterozygotes (Fig. 3). Although the mean and 95% confidence intervals for G6PD activity are well below normal, the range of values extends above the mean for normal G6PD. These values appear to represent a normal distribution between 0 and 100% of normal, as random Lyonization would yield. About half of these females may have more than 50% of their RBC populations as fully deficient as seen among Vanua Lava male hemizygotes. They also would perhaps prove vulnerable to a threatening AHA with primaquine anti-relapse therapy.

The females heterozygous for Viangchan exhibited a significantly narrower and lower range of G6PD activity. The reasons for this difference are unclear, but the finding bears important clinical, public health and genetic implications worthy of follow up investigation. Non-random Lyonization somehow favoring the mutant X chromosome would perhaps explain the observations [41]. Although Lyonization is random as a whole organism, non-random or skewed lyonization does occur in individual cells or tissues [41][42]. Regardless of mechanism, the findings from Sumba suggest that Viangchan females may be intrinsically more vulnerable to primaquine therapy than females heterozygous for Vanua Lava. Chatham appears intermediate between the two (Fig. 3).

Another important phenomenon observed in the survey at Sumba is also evident in Fig. 3. The mean value of those expressing normal G6PD activity was sharply skewed upwards. Fig. 4 illustrates the basis of this skewing. Below a hemoglobin level of 10g/dL, G6PD activity rose sharply in linear fashion with decreasing hemoglobin levels. In other words, anemia appeared to directly impact G6PD activity in a quantitative manner. There are at least two possible explanations for this observation: 1) reticulocytemia among the anemic; or 2) mathematical treatment of G6PD activity estimates based on hemoglobin levels systematically biasing the estimate. Reticulocytes express the highest levels of G6PD activity, and that decays naturally as RBC age [43][44]. That subpopulation being overrepresented among the anemic would push G6PD values upwards. Alternatively, this effect may simply be due to the hemoglobin concentration being the denominator of the observed enzymatic activity for the per g/dL hemoglobin estimation. In other words, actual enzyme activity may be artificially inflated when the concentration of Hb is naturally low. This phenomenon may obscure truly deficient G6PD by falsely reporting normal activity. Kinetic parameters of G6PD activity may provide deeper insights into relative vulnerability to AHA by primaquine. NADPH competitively inhibits G6PD in the phosphate pentose shunt pathway. Further, G6PD is very strictly regulated in the cytosol where inhibition by NADPH becomes stronger with lower concentration of substrate, glucose-6-phosphate. Therefore, a high K_m_ for NADP or low Ki for NADPH would more strongly inhibit G6PD, rendering the enzyme far less functional, whereas low K_m_ for NADP (high Ki for NADPH) would result in diminished NADPH inhibition and more active G6PD [26]. These parameters obviously bear upon realized G6PD activity under conditions of oxidative stress and threat of RBC destruction by it. Our findings with G6PD kinetics hint at possibly important distinctions among the variants. Whereas Vanua Lava and Viangchan hemi/homozygotes had markedly low Km for NADP+ compared to normal (Table 3), Chatham variants were higher than normal. How this may bear upon primaquine sensitivity bears further investigation. The kinetics studies were hampered by relatively low numbers of successfully analyzed samples, and no firm conclusions may be drawn from them. The kinetics studies were severely limited by the relatively low volume of blood collected and the laborious and expensive methods of analysis required within a short span of time. Pioneering G6PD scientist Ernest Beutler expressed that low residual enzyme in a deficient sample may be a product of transportation of samples, extraction steps, length of sample storage or estimations during the biochemical parameters themselves [45]. We accept that such may have occured in our experiments, but the kinetics trends among variants and accordance with the quantitative lysate measurements offers reassurance. The sample for kinetics was not controlled for reticulocyte counts or for the presence of other inherited blood disorder common in Sumba, like Southeast Ovalocytosis, hemoglobin E, and other thalassemias. The possibility of an important sampling bias in the kinetics analyses may not be ruled out. We considered these analyses exploratory in nature rather than conclusive in findings.

In summary, our large survey of G6PDd in western Sumba revealed the disorder to be prevalent, diverse and severe, informing the assessment of risk versus benefit with primaquine therapy, both with and without G6PDd screening. We observed possibly important differences in G6PD expression among heterozygous variants, and revealed anemia as the basis of G6PD measurements skewed far above normal. This epidemiological evaluation of G6PDd at Sumba highlights the complexity of this disorder in light of primaquine safety with therapy against relapse of vivax malaria.

## Supporting Information

S1 Checklist STROBE checklist. (DOCX)
